# Clinical Implications of Paternal Age in Assisted Reproduction: Integrating Sperm Epigenetic Evidence

**DOI:** 10.3390/jcm15041324

**Published:** 2026-02-07

**Authors:** Dimitrios Diamantidis, Konstantinos Nikolettos, Nektaria Kritsotaki, Angeliki Tiptiri-Kourpeti, Nikolaos Nikolettos, Georgios Tsakaldimis, Stilianos Giannakopoulos, Christos Kalaitzis

**Affiliations:** 1 Department of Urology, Democritus University of Thrace, 68100 Alexandroupolis, Greece; 2 Department of Gynaecological Oncology, Maidstone and Tunbridge Wells NHS Trust, Maidstone ME14 1FZ, UK; 3 Department of Obstetrics and Gynecology, Democritus University of Thrace, 68100 Alexandroupolis, Greece; 4 Genesis Athens–Thrace Medically Assisted Reproduction Unit, 68100 Alexandroupolis, Greece; 5 Laboratory of Reproductive Physiology–IVF, Democritus University of Thrace, 68100 Alexandroupolis, Greece

**Keywords:** advanced paternal age, sperm epigenome, assisted reproduction, DNA methylation, nucleosome retention, small non-coding RNA, IVF/ICSI, embryo morphokinetics, miscarriage, counseling

## Abstract

**Background:** Advanced paternal age is increasingly encountered in assisted reproduction as parenthood is deferred. The clinical question is whether paternal age from about 40 to 45 years and older affects embryo development or outcomes, and to what extent any effect relates to the sperm epigenome. **Methods:** This narrative review synthesized PubMed-indexed evidence on sperm aging biology, including DNA methylation, chromatin packaging and nucleosome retention, small non-coding RNAs, telomere dynamics, DNA fragmentation, and oxidative and mitochondrial stress, and their potential clinical impact on assisted reproduction outcomes. **Results:** Maternal age remains the principal determinant of embryo aneuploidy. After multivariable adjustment, independent paternal-age effects on fertilization, blastocyst formation, and preimplantation genetic testing for aneuploidy are small or not detected. At very advanced paternal ages near or above 50 years, some studies report higher miscarriage and lower live birth, without a consistent change in early embryo morphology. Aging in men is linked to higher DNA fragmentation and oxidative and mitochondrial signatures, together with reproducible sperm-epigenome changes, including age-linked DNA methylation, altered histone retention, and small-RNA shifts. These molecular findings support modest intergenerational influences on early development, while stable transgenerational inheritance in humans is not supported. **Conclusions:** Advanced paternal age should be regarded as a risk modifier rather than a primary driver of preimplantation failure. Counseling should emphasize realistic effect sizes and the predominance of maternal age. Laboratory workflows should minimize oxidative stress. Selective DNA-fragmentation testing may be appropriate in recurrent ART failure or recurrent loss. Sperm-epigenome assays remain investigational and should undergo prospective, standardized validation before use in routine care.

## 1. Introduction

Advanced paternal age (APA) is increasingly encountered as parenthood is deferred, and the use of assisted reproductive technologies (ARTs) expands. A recurring clinical question is whether paternal age ≥ 40–45 years affects embryo competence, aneuploidy, implantation, or offspring health. Maternal age remains the principal driver of chromosomal risk. Contemporary evidence indicates that male aging is accompanied by biological remodeling of the sperm cell, not merely minor shifts in routine semen parameters [1,2]. Aging is associated with lower motility and higher DNA fragmentation index (DFI), yet independent links to lower ART success are weak once maternal factors are controlled [3].

Sperm delivers more than the paternal genome. It carries structured chromatin and epigenetic information (protamine–histone balance, nucleosome retention, DNA methylation, small non-coding RNAs), and disturbances across these layers have been linked to fertilization, cleavage kinetics, blastocyst formation, and implantation [4,5,6]. Age-related methylation drift, telomere changes, and rising DNA fragmentation comprise an aging signature. Multi-CpG “clocks” track chronological age and have been variably related to reproductive timing [1,7,8,9]. Mechanistic work links paternal DNA hypomethylation to increased nucleosome retention in sperm and subsequent gains of H3K4me3 (trimethylation of histone H3 at lysine 4), a chromatin mark of transcriptionally active promoters, at specific paternal regulatory regions in early embryos. This supports a methylation-to-chromatin-to-embryo axis relevant to zygotic genome activation (ZGA) [10]. In parallel, defined sperm sncRNA signatures have been associated with embryo competence [5,11,12]. Human embryo datasets indicate that the preimplantation period is a sensitive window for epigenetic reprogramming, during which imprinting control regions (ICRs) can exhibit measurable deviations in DNA methylation maintenance. This provides biological plausibility for intergenerational influences in ART, while also underscoring that embryo-stage signals require cautious interpretation because they reflect both parental inputs and culture-related factors [13,14].

Clinically, once maternal age and key laboratory variables are accounted for, the independent contribution of APA to fertilization rate, blastulation, morphokinetics, and preimplantation genetic testing for aneuploidy (PGT-A) outcomes is typically modest, with effect sizes that are smaller than those attributable to oocyte age. Consistently, donor-oocyte studies, where oocyte age is held constant, rarely demonstrate robust paternal-age-driven differences in blastulation or embryo aneuploidy [2,15]. Global sperm 5-methylcytosine (5mC) relates only weakly to ICSI outcomes, indicating that locus-specific or pathway-level signals are more informative than coarse global metrics [16]. Notably, paternal age-associated methylation changes identified at CpG sites in human sperm do not persist through germline reprogramming in offspring tissues, which argues against stable transgenerational methylation inheritance [17]. Overall, the evidence supports subtle intergenerational mechanisms (epigenetic drift, chromatin bookmarking, oxidative and mitochondrial stress) rather than large paternal effects on aneuploidy [18,19,20].

This narrative review integrates APA and sperm epigenetics within a clinically oriented framework. Evidence across embryo-stage endpoints, including fertilization, morphokinetics, blastulation, preimplantation genetic testing for aneuploidy (PGT-A), and available early embryo omics, is synthesized alongside donor-oocyte contrasts (where oocyte age is held constant) to better contextualize paternal contributions [21,22,23,24]. These data are used to inform counseling and laboratory workflow considerations, particularly in relation to sperm DNA integrity and oxidative stress during sample preparation, with attention to realistic effect sizes in men aged ≥40–45 years and potentially modifiable factors such as metabolic health, smoking, pollutant exposure, and oxidative-stress minimisation during sperm handling. Finally, embryo-stage observations are related to offspring outcomes, distinguishing the established de novo mutation pathway from epigenetic hypotheses that remain under investigation and may require embryo- or placenta-level confirmation in humans [25,26,27,28].

## 2. Materials and Methods

### 2.1. Scope

This review aims to summarize current evidence on advanced paternal age (APA) in assisted reproduction, with a focus on how sperm aging biology, including epigenetic and other molecular alterations, relates to IVF/ICSI outcomes and clinical counseling.

### 2.2. Literature Search

A targeted and structured search of PubMed was conducted up to November 2025. The core search strategy combined terms for paternal age, assisted reproduction, and sperm biology, including: “advanced paternal age”, “paternal age”, “older fathers”, “assisted reproduction”, “IVF”, “ICSI”, “ART”, “sperm”, “semen”, “epigenetic”, “DNA methylation”, “chromatin”, “nucleosome retention”, “histone”, “small non-coding RNA”, “miRNA”, “sncRNA”, “DNA fragmentation” and “oxidative stress”. Only peer-reviewed articles in English were considered. Additional relevant articles were identified by screening reference lists of key primary studies and recent reviews, with iterative cross-checking to ensure that all major and frequently cited studies on APA in ART and sperm biology were captured.

### 2.3. Eligibility Criteria

We included studies reporting ART-related endpoints in relation to paternal age, specifically fertilization, embryo morphokinetics, blastocyst formation, PGT-A aneuploidy, clinical pregnancy, miscarriage, and live birth, with priority given to large single-center or multicenter cohorts, donor-oocyte studies that constrain oocyte age, and systematic reviews or meta-analyses where available. We also incorporated experimental and translational work on sperm aging biology that investigated associations between paternal age and DNA methylation, chromatin organization and nucleosome retention, histone modifications, small non-coding RNAs, telomere dynamics, DNA fragmentation, mitochondrial function, and oxidative stress, particularly when these alterations were related to embryo-stage readouts. Eligible designs comprised prospective and retrospective observational cohorts, registry-based analyses, and translational studies with clearly described IVF/ICSI or embryo-assessment protocols. Case reports, very small series, and studies limited to natural conception without direct relevance to ART practice were not retained.

### 2.4. Study Synthesis

Identified records were screened on title, abstract, and, when needed, full text to determine eligibility for this review. The evidence was then synthesized narratively, with particular attention to study design, sample size, adjustment for maternal age and key confounders, clarity of ART protocols, and definition of outcomes. No quantitative meta-analysis or formal risk-of-bias scoring tool was applied.

## 3. Biology of Paternal Aging in Sperm

### 3.1. DNA Methylation

Across independent human studies, advancing paternal age associates with germline-specific methylation drift in sperm, with whole-genome/capture profiling in rigorously screened men identifying >200 age-DMRs, rising DNA fragmentation, and divergent telomere dynamics [1]. RRBS reports >1500 mainly hypomethylated regions with developmental enrichment, reinforcing a non-random, sperm-specific aging program [29]. Higher-resolution array/capture studies detect tens of thousands of age-linked CpGs and enable sperm clocks, with notable DMR clusters near PPARGC1A and RBFOX1 [7,8,29,30,31].

Clinical studies report that couples in which the male partner’s sperm shows an older epigenetic age have a longer time to pregnancy and slightly shorter gestation [8]. Beyond time to pregnancy, higher paternal age has been associated with slower embryo development and lower live-birth rates. Methylome profiling identifies ~1700 age-associated CpGs and ~1100 regions enriched for embryonic development and neurobiological pathways, supporting sperm DNA methylation as a candidate mediator in ART [32]. Additional human studies link advancing age with higher global sperm methylation, increased DNA fragmentation, chromatin decondensation, and poorer ICSI outcomes, aligning with broader observations in APA research [8,32,33,34].

Mechanistically, spermatogenic DNA methylation is set by the DNA methyltransferases DNMT1, DNMT3A, DNMT3B, DNMT3C, and the cofactor DNMT3L, with locus-specific control at imprinted regions such as H19 and MEST and repression of transposable elements through DNMT3C and PIWI-interacting RNA (piRNA) pathways. Disruption of these modules is linked to male infertility phenotypes and outlines how age-related drift could intersect programmed methylation states [35]. Beyond global proxies such as long interspersed nuclear element 1 (LINE-1), locus-level assays indicate that the sperm ribosomal DNA (rDNA) promoter is age sensitive. In one analysis, promoter methylation rose markedly with paternal age by linear regression, *p* < 0.0001, whereas LINE-1 showed no age association in the same sample set, supporting the view that targeted loci are more informative than global surrogates [36].

Critically, although sperm age-associated methylation marks are robust and reproducible, embryo and offspring tissues do not retain these DMR patterns after post-fertilization reprogramming. Therefore, consequences should be viewed as intergenerational and reprogramming-sensitive, not stable transgenerational inheritance [17,20,30].

### 3.2. Histone Landscape and Nucleosome Retention

A mechanistic bridge between sperm DNA methylation and embryonic chromatin comes from nucleosome-retention studies. In mice, experimentally reduced sperm methylation increases nucleosome retention and permits de novo H3K4me3 at paternal promoters, subtly shifting ZGA timing and amplitude [10]. KDM5B (JARID1B) fine-tunes ZGA onset, placing H3K4 writers/erasers downstream of methylation-driven changes in retention and reinforcing a methylation-to-nucleosome-to-H3K4 pathway [21,37,38]. Retained sperm histones at CpG-rich promoters/regulatory elements may serve as epigenetic bookmarks, complementing RNA-mediated routes [10,21]. The histone-to-protamine transition shapes accessibility/compaction. Disruption shifts retention at developmental loci and underlies defective packaging [35]. Clinically, an imbalanced P1:P2 and increased histone retention are associated with poorer embryo morphology and reduced blastocyst yield, plausibly via premature chromosomal condensation, impaired paternal-genome decondensation, and asynchronous zygotic cell-cycle progression [4].

Human sperm exhibits nonrandom nucleosome retention at developmentally important loci, including imprinted domains, microRNA clusters, and HOX genes, supporting delivery of a targeted histone cargo to the zygote [39]. Follow-up profiling links altered retention and histone post-translational modifications at these loci to subfertility [40]. Calibrated ChIP-seq shows that a subset of H3 methyl marks is retained at consistent genomic positions in sperm and can persist through early cleavage divisions, marking developmental genes whose expression is perturbed when histone methylation is experimentally reduced [41].

Embryo-level readouts converge on paternal H3K4 methylation. MLL3- and MLL4-dependent paternal H3K4me1 support the minor ZGA wave in the male pronucleus [37], and paternal H3K4me3 can evade protamine-mediated eviction and be detected up to implantation, with enrichment at paternally expressed genes [38]. Recent syntheses place retained nucleosomes and their modifications at promoters, enhancers, and super enhancers, often colocalized with architectural proteins such as CTCF and cohesin, providing plausible anchor points through which paternal chromatin influences early regulatory topology [42]. Diagnostic assays capture complementary facets of packaging and damage. CMA3, aniline blue, and toluidine blue assess chromatin compaction. SCSA and DFI quantify strand integrity. Clinical interpretability and interchangeability remain limited without standardized pipelines [13].

Clinically, physiological aging likely produces subtle versions of the experimental effects seen in models, but convergent evidence supports a methylation, nucleosome, H3K4 route through which paternal aging can modulate the chromatin cargo delivered to the zygote and fine-tune ZGA kinetics [10,21,37,38,39,40,41].

### 3.3. Small RNAs (miRNA/tsRNA/piRNA)

Across studies, human sperm show a stable sncRNA set and a variable layer that shifts with age and exposures; this framework explains why some proposed biomarkers have proven hard to reproduce and which RNAs are more likely to be stable carriers versus modifiable signals [5]. Single-center work that combined micromanipulation of about 1500 individual sperm per sample with small RNA sequencing and RT qPCR validation reported that miR-15b-5p, miR-19a-5p, and miR-20a-5p associate negatively with sperm count, progressive motility, and morphology. Higher expression also tracked with poorer embryo grades and lower β hCG and live birth rates. Area under the curve values were approximately 0.71 to 0.76, and a combined model reached approximately 0.75 [43]. In human IVF studies, specific sperm miRNAs, notably let-7g and miR-30d, associate positively with embryo quality, whereas subsets of 28S rRNAs show inverse associations. Composite small RNA panels provide clinically informative discrimination [11]. Beyond miRNAs, clinical syntheses indicate that piRNAs and tsRNAs correlate with semen quality and fertilization metrics [44]. Foundational studies confirm that mature human sperm contain robust 18S and 28S rRNA-derived fragments [45,46].

Mechanistic work in mammals indicates that tsRNAs can transmit paternal environmental and aging signals. Dietary changes remodel sperm tsRNA profiles, and RNA modifications, and purified tsRNA fractions can reproduce metabolic phenotypes after zygotic microinjection [47,48]. During epididymal transit, soma-to-germline transfer supplies sperm with miRNAs, tsRNAs, and rsRNAs, creating a route for age and lifestyle influences to modify the sncRNA payload before ejaculation [48,49,50,51]. Age-linked miRNAs offer a plausible connection to DNA-damage responses and early embryo timing. Overall, current data point to a conserved sncRNA scaffold, an exposure-responsive peripheral layer, and candidate mediators through which paternal aging may fine-tune cleavage dynamics, ZGA timing, and early lineage allocation [3,5,11,12,21,31,44,45,46,47,48,49,50,51,52].

### 3.4. Telomeres, DNA Fragmentation (DFI), and mtDNA/Oxidative Stress

In rigorously screened healthy men, leukocyte telomere length (LTL) shortens with age while sperm telomere length (STL) increases, consistent with germline telomerase activity; higher paternal age at conception associates with longer offspring LTL, indicating biological relevance of STL dynamics [1,53]. In the same cohort, DFI rose with age, with many men > 65 years exceeding clinical cut-offs. Meta-analyses link higher sperm DNA fragmentation to greater miscarriage risk and reduced fecundability, though effect sizes vary by assay/study design [54,55]. Because oocytes can repair only limited paternal DNA damage, selective DFI testing is reasonable in RPL or repeated ART failure [56].

Oxidative stress (OS) and mitochondrial dysfunction are proximate drivers of sperm DNA damage: excess ROS promotes strand breaks (↑DFI), lipid peroxidation, and protein oxidation, impairing fertilization and embryo development. Mature sperm are particularly vulnerable to ROS because they have limited cytosolic antioxidant defenses and constrained post-meiotic DNA repair capacity, making oxidative injury more likely to manifest as functional deficits rather than recoverable molecular lesions. In clinical datasets, this “oxidative-stress axis” is typically operationalized through DFI, progressive motility, and mitochondrial readouts such as sperm mtDNA copy number, which capture the downstream footprint of oxidative injury without requiring detailed pathway-level interpretation [51,57,58]. Human studies consistently associate higher sperm mtDNA copy number with abnormal semen parameters and reduced motility/concentration, often interpreted as a compensatory yet dysfunctional response [59,60,61]. Mechanistic links connect OS to early embryo events: oxidative damage in sperm relates to impaired active DNA demethylation in the paternal pronucleus and poorer IVF outcomes [62,63].

ART handling can amplify ROS exposure: longer or repeated centrifugation increases ROS and harms sperm function, reducing spin duration/rounds, and considering microfluidic or cushioned protocols mitigates iatrogenic OS [64,65,66]. Overall, paternal aging yields a composite profile, longer STL, higher DFI, and OS/mitochondrial signatures that can depress fertilization potential and compromise early development. The converging evidence supports ROS-minimizing laboratory workflows and selective DFI for clinical triage, while research should refine mtDNA-/OS-linked biomarkers with standardized assays and thresholds [57].

### 3.5. From Marks to Function: Pathways and Plausible Mechanisms

Human sperm age-DMRs show non-random topology. CpGs that lose methylation with age cluster near open chromatin H3K27me3-marked developmental promoters, whereas gains shift toward compact chromatin; locus clustering near PGC-1α and RBFOX1 aligns with neuronal/developmental programs [1,7]. Integrative syntheses map age-linked DNA methylation and small-RNA shifts within chromatin-remodeling frameworks [21,31,67]. A causal bridge is demonstrated in mice: reducing sperm DNA methylation increases nucleosome retention and permits de novo H3K4me3 at paternal promoters, subtly shifting ZGA timing; paternal H3K4 is required for the minor ZGA wave, consistent with a methylation, nucleosome, H3K4 route [10,37,38].

In human IVF, sperm-borne miRNAs associate positively with embryo quality, whereas subsets of 28S-derived rsRNAs show inverse associations; composite small-RNA panels are clinically informative, and miR-125a-5p links aging to DNA damage and stage-specific embryo delays via RBM38–p53 [11,12]. Despite robust aging signatures in sperm, age-associated CpG patterns do not persist after post-fertilization reprogramming in human offspring tissues, framing effects as intergenerational rather than fixed across generations [17]. Comparative RRBS underscores species specificity; marmoset age-DM TSS tend to gain methylation with age, whereas human sites predominantly lose methylation, with no orthologous overlap, warranting caution in extrapolation [68]. Overall, three linked layers—DNA methylation at chromatin-primed developmental loci, retained nucleosomes/H3K4 dynamics after fertilization, and sncRNAs with embryo correlates—offer a plausible, modest-effect framework by which paternal aging can tune ZGA and early lineage priming within the bounds of embryonic reprogramming capacity [1,7,10,11,17,21,31,37]. Key APA-linked changes in sperm, their assays, representative loci, and embryo-stage links are summarized in Table 1.

## 4. Preimplantation Readouts Linking Sperm to Embryo

### 4.1. From Fertilization to ZGA: Paternal Contributions and Candidate Regulators

Human sperm seems to contribute more than the paternal genome [69,70]. It delivers functional centrioles, the oocyte-activating factor phospholipase C zeta (PLCζ), a packaged chromatin landscape with retained nucleosomes and defined histone marks, and a small-RNA cargo capable of early post-fertilization signaling [6,71]. Human studies show that sperm carry two remodeled, functional centrioles that reconstitute the zygotic centrosome and support pronuclear migration and early cleavages [72,73]. For oocyte activation, convergent clinical and experimental evidence indicates that PLCζ is the principal sperm factor that drives Ca^2+^ oscillations. PLCZ1 defects are linked to fertilization failure and inform assisted oocyte activation strategies [51,74].

A mechanistic methylation of chromatin to the embryonic axis is defined in the mouse. Reducing sperm DNA methylation increases nucleosome retention and renders paternal alleles permissive for de novo H3K4me3 deposition in early embryos, which subtly shifts the timing of ZGA [10]. In parallel, paternal H3K4 methylation in the male pronucleus is required for the minor ZGA wave, supporting a causal role for paternal chromatin in initiating embryonic transcription. Additional embryo-stage data indicate that subsets of paternal histone marks persist into cleavage stages and influence early gene regulation. Calibrated embryo assays implicate paternal H3K4 and the paternal H2AK119ub1 signal as necessary for development, reinforcing a chromatin-based paternal contribution [37].

RNA-mediated routes operate alongside chromatin bookmarking. In IVF datasets, specific sperm-borne miRNAs such as let-7g and miR-30d correlate positively with embryo quality, whereas subsets of rRNA-derived small RNAs show inverse associations. Composite small-RNA panels classify embryo quality with clinically relevant accuracy [11]. A cross-population atlas distinguishes a conserved core sncRNAome from a peripheral layer that is sensitive to age and environment, clarifying which RNAs are plausible stable carriers versus modifiable signals [5]. Age-linked miR-125a-5p increases with paternal aging, augments DNA damage, perturbs morula/blastocyst progression, and acts via RBM38–p53, nominating a concrete pathway by which age-shifted small RNAs can influence early development [12,71].

### 4.2. Embryo-Level Biomarkers (PGT-A, Morphokinetics, Embryo-Omics)

Across IVF and ICSI datasets, the independent effect of paternal age on fertilization rate, early cleavage kinetics, and blastocyst formation/blastulation is generally small to null once maternal age and key protocol/laboratory covariates such as insemination method, culture conditions, and workflow are controlled. Signals that appear in univariate analyses typically attenuate after adjustment. Representative datasets and syntheses report similar conclusions [15,75,76]. Time-lapse evidence is limited and heterogeneous. Reported paternal-age associations with intervals such as t2 to t5, s2, and cc2 are inconsistent and often underpowered. Large datasets indicate that non-age confounders, including laboratory workflow, insemination method, culture media, and maternal factors, explain much of the variance in morphokinetics [77]. In a prospective time-lapse series using ICSI and IMSI with 1210 embryos from 151 couples, paternal age and BMI were associated with subtle shifts from tPNa to t4 through t6 after adjustment. Sperm concentration showed less consistent effects, and SDF was not significant in the adjusted model [78]. A small IVF-only study reported that sperm rDNA-promoter methylation correlated marginally with 2PN fertilization overall and significantly at specific CpGs such as CpG_10, while day-3 quality and clinical pregnancy did not associate. This pattern supports locus-specific paternal signals at fertilization rather than broad embryo-quality shifts [69].

Aneuploidy risk is dominated by maternal age. In donor-oocyte designs and adjusted couple-level analyses, most studies find no meaningful independent association between paternal age and embryo aneuploidy. Some recent retrospective series report weak or model-dependent trends, but these are not universal and need replication with rigorous adjustment. Overall, paternal age rarely alters PGT-A results once maternal age is accounted for [15,75,76]. Direct embryo methylome or transcriptome readouts that isolate paternal age remain sparse. Sperm–embryo correlation studies indicate that defined paternal small RNA or methylation signatures track with embryo competence or early transcriptional states. Non-invasive PGT-A approaches that analyze cell-free DNA from spent media are expanding embryo-omics, but current work focuses on analytical performance and workflow rather than paternal-age effects [79].

Across clinically used preimplantation endpoints, including fertilization, blastulation, PGT-A, and morphokinetics, paternal age exerts at most modest independent effects. Where associations are detected, they usually weaken after controlling for maternal age and laboratory variables. Mechanistic readouts from chromatin and sncRNA provide biological plausibility for small paternal influences on early development, but direct embryo-level validation that cleanly isolates paternal age remains limited [15,77]. Adjusted preimplantation outcomes by paternal age and clinical setting are shown in Table 2.

### 4.3. Donor-Egg vs. Autologous Models

With oocyte age held essentially constant, large adjusted series and an individual patient data meta-analysis show no independent association between paternal age and embryo aneuploidy. Blastulation likewise shows no consistent paternal age signal. When a signal appears, it is small and model dependent, with a modest reduction in fertilization rate and a slight rise in segmental abnormalities only at very advanced paternal ages, approximately 50 years or older [15,75,76].

Strong collinearity between paternal and maternal age and heterogeneity in clinical and laboratory practice complicate inference. In contemporary series, apparent associations between paternal age and embryo kinetics or aneuploidy generally attenuate after multivariable adjustment, with maternal age remaining the dominant driver [52,82].

Donor oocyte evidence supports that paternal age is not a major driver of preimplantation failure modes in contemporary ART. If paternal age effects exist, they are likely subtle and context dependent, and mechanistically more consistent with chromatin and small RNA pathways than with large shifts in embryo aneuploidy [52,75,76].

### 4.4. Imprinting and Placental Signals at the Preimplantation Interface

In ART embryos, imprinting errors are common and largely stochastic, occurring even in morphologically top-grade embryos and without excess risk from extended versus short culture. Morphology, therefore, does not guarantee imprint fidelity [14]. On the paternal side, age-linked sperm DMRs can map near imprinting circuitry. For example, locus-limited associations at FOXK1 and KCNA7 support proximity effects rather than genome-wide imprint disruption [70]. In blastocysts from fathers ≥ 50 years, genome-scale methylome shifts have been reported in both ICM and TE with enrichment for neurodevelopmental pathways; imprinted loci are over-represented, and KCNQ1OT1 hypermethylation in the ICM has been validated, whereas TE transcriptomes show no pathway-level disruption, suggesting a fetal-lineage-skewed signal rather than a broad placental transcriptomic failure [23]. Crucially, age-associated CpG patterns identified in sperm do not persist in offspring tissues after reprogramming, framing effects as intergenerational and reprogramming-sensitive, not fixed across generations [17].

Human embryo stage imprinting data that isolate paternal age remain limited. Animal studies indicate APA-linked perturbations of placental growth and imprinted gene expression [23]. A recent human placental study reports small, locus-limited paternal age-associated methylation shifts with enrichment for neurodevelopmental genes, which suggests that the placenta may serve as a sensitive readout of paternal signals without broad imprint failure. ART and infertility-related factors can also alter placental epigenetics, which complicates causal attribution [83].

These observations do not justify changes from the clinical aspect to first-line ART protocols based on paternal age alone. They support balanced counseling: (i) imprinting errors can arise in ART irrespective of paternal age and should be considered background/stochastic at the embryo stage [14], (ii) paternal-age signals, where present, appear locus-limited and modest, more consistent with chromatin/sncRNA pathways than with large shifts in aneuploidy or global imprint failure [23,70], and (iii) potential placental readouts of paternal age are subtle, and ART/infertility factors themselves can influence placental epigenetics, complicating attribution [83]. Embryo-stage imprinting and methylome readouts relevant to APA are summarized in Table 3.

## 5. Clinical Evidence in ART

### 5.1. Semen Parameters vs. the Sperm Epigenome: When They Disagree, What “Counts” Clinically?

Standard semen parameters, such as concentration, motility, and morphology, show at most mild and inconsistent shifts with advancing APA, whereas DNA fragmentation (DFI) rises more consistently in parallel with oxidative stress [1,20]. Sperm viability (vitality) should also be considered, because it shows a subtle but significant age-related decline in healthy men, alongside reduced progressive motility [1]. Across IVF and ICSI datasets, after adjustment for maternal age and laboratory factors, conventional semen parameters show generally only a weak clinical signal for downstream outcomes [2,3,71]. Large single-center data sets, approximately 6800 semen tests and 1200 ART cycles, confirm declining motility and higher DFI with age, yet no corresponding decline in ART outcomes was observed [3].

These observations support a clinically relevant pattern: age-related deterioration in sperm functional integrity (particularly DNA fragmentation and, to a lesser extent, motility/viability) can be measurable even when conventional semen measures remain within reference ranges. In donor-oocyte ICSI cycles, where oocyte age is constrained, recent evidence indicates that advanced paternal age (≥45 years) can be accompanied by reduced blastulation and lower implantation/live birth when conventional processing is used. At the same time, microfluidic selection that enriches for lower-fragmentation sperm is associated with improved blastulation, implantation, and live birth in older men [84]. Mechanistically, oxidative stress is a plausible upstream driver of DNA damage and may contribute to this “functional” decline, with potential consequences for early embryonic development when oocyte repair capacity is exceeded [85].

When routine metrics are unrevealing, locus-specific methylation can still carry aging information. For example, rDNA promoter methylation increases with paternal age even as LINE-1 remains unchanged, illustrating why global 5mC can underperform compared with biologically anchored loci [36]. Time-lapse analyses likewise suggest that conventional semen metrics may miss subtle paternal influences detectable in embryo kinetics, with age and BMI effects on tPNa through t6, while SDF showed no adjusted association with morphokinetics in that series [78].

Reviews synthesize that protamine P1 to P2 skew, excess histone retention, elevated DFI, and sperm aneuploidy can track with embryo quality decrements even when count and motility are acceptable, although findings are assay dependent [4]. Multiple human studies show age-linked remodeling of the sperm epigenome. These include DNA methylation age DMRs and epigenetic clocks, shifts in histone retention and marks, and remodeling of the small RNA cargo [1,5,7,10,12]. Some studies link older sperm epigenetic age to longer time to pregnancy and subtle decrements in early developmental readouts despite normal semen parameters [8,9]. In contrast, global sperm 5mC shows weak or null associations with ICSI outcomes under controlled conditions, underscoring the need for locus- or pathway-specific profiling rather than coarse global metrics [16]. On the RNA side, IVF studies associate defined paternal miRNAs and rsRNAs with embryo quality, and mechanistic work ties miR-125a-5p to p53 pathway dysregulation in aging, linked to DNA damage [5,12].

A large WHO-2021–based analysis of IVF/PGT-A cycles provides a clinically useful bridge between semen metrics and embryo outcomes. Semen quality did not independently influence euploidy or implantation, which is compatible with early embryo arrest before blastulation or oocyte-mediated correction prior to embryonic genome activation. Maternal age remained the dominant determinant of euploid blastocyst rate, whereas semen parameters below the 5th percentile, particularly low motility and combined deficits in concentration and morphology, were associated with reduced euploid blastocyst formation. Both obstructive and non-obstructive azoospermia showed similarly lower euploid blastocyst rates, underscoring that when semen quality is severely impaired, a measurable impact on embryo competence can still emerge despite oocyte buffering [86].

In a recent single-center donor oocyte study with 320 transfers and donors aged 35 years or younger, paternal age showed a graded association with clinical endpoints. Adjusted odds of live birth were about 0.50 for 46 to 50 years and 0.21 with a 95% confidence interval of 0.11 to 0.38 for 51 years or older. Miscarriage risk was higher with an adjusted odds ratio of 2.08 compared with those 35 years or younger, while fertilization and blastocyst rates did not differ across age groups [80]. These findings are compatible with a post-fertilization influence of paternal aging rather than effects on early morphological milestones.

When semen analysis and epigenomic signals appear to disagree, current data suggest that epigenomic/small-RNA signatures may sit closer to the machinery influencing early development, but validated clinical thresholds are not established, and platform heterogeneity is substantial [5,10,12,21]. Semen analysis remains the first-line assessment. DFI can be considered in selected scenarios such as unexplained ART failure, recurrent early loss, or suspected high ROS. Epigenomic assays, including methylation signatures and sperm small RNA panels, are investigational and should be interpreted with caution. None is validated as a stand-alone surrogate of embryo competence.

When semen quality is severely impaired, such as very low motility, combined deficits in concentration and morphology, or azoospermia, euploid blastocyst yield and live birth can be reduced despite oocyte buffering, particularly in donor-oocyte cycles and in young-maternal-age strata [3,75,76,80]. Within normal or mildly impaired semen ranges, paternal-age-related deterioration in sperm characteristics tends to translate into small, often context-specific differences in fertilization, implantation, or miscarriage rather than large, universal shifts in ART success [2,3,78,81,87]. In practice, semen quality and DFI may worsen with age, while ART outcomes remain similar across paternal-age strata, implying substantial oocyte-stage and laboratory buffering [3].

### 5.2. IVF/ICSI Outcomes: Fertilization, Blastocyst Formation, Aneuploidy, Clinical Pregnancy/Live Birth

After adjustment for maternal age and laboratory covariates, the independent effect of paternal age on fertilization and blastocyst formation is small and often non-significant. Time-lapse morphokinetic signals are inconsistent and readily confounded by oocyte age and workflow [2,71]. Maternal age remains the dominant driver of embryo aneuploidy. Most adjusted analyses detect no meaningful paternal-age effect on PGT-A [2,76]. Large single-center datasets report no significant differences by paternal-age strata in insemination-method distribution, usable-embryo rate, blastocyst availability, or clinical and live-birth rates [3].

#### 5.2.1. Fertilization and Early Cleavage

After controlling for key clinical and laboratory factors, little independent effect of paternal age remains [2,71]. Time-lapse studies report small and heterogeneous paternal-age associations in specific intervals, most commonly early cleavage timings, cell-cycle measures, and synchronicity, with direction and magnitude varying across studies. Much of this variability appears driven by non-age confounders, including maternal factors, insemination method, culture media, and laboratory workflow, which frequently explain a larger share of morphokinetic variance than paternal age itself [2,77,78,81]. In a time-lapse–enabled program, paternal age worsened semen parameters but did not independently reduce fertilization, blastocyst development, FET gestation rates, or iDAScore. Maternal age correlated with slower kinetics, lower scores, and lower gestation rates [81]. Notably, within the same program, fertilization and blastocyst rates remained similar across paternal-age groups even when embryo-lineage methylomes were perturbed [23]. If fertilization dips in older fathers, first interrogate oocyte age and lab workflow; use assisted oocyte activation only when indicated by the clinical picture rather than paternal age [2,71].

Additional IVF cohorts refine these observations. In non–male-factor IVF cycles, paternal age ≥ 40 years was associated with lower implantation and reduced clinical pregnancy rates in frozen embryo transfer cycles, despite similar fertilization, high-quality embryo proportions, and blastocyst formation across age groups [88]. This pattern is consistent with a post-zygotic influence of paternal aging, acting downstream of fertilization. In unexplained infertility, paternal age > 35 years was linked to lower fertilization and fewer cleaved embryos, while clinical pregnancy, miscarriage, and live-birth rates remained similar after controlling for maternal factors and semen processing [89]. Collectively, these data indicate that paternal age can modestly affect fertilization efficiency and implantation potential without imposing large, consistent shifts in early cleavage or blastocyst yield.

#### 5.2.2. Blastocyst Formation and Embryo Usability

Across modern lab conditions, blastulation is generally neutral with respect to paternal age after adjustment [2,3,71,81]. When small differences are observed, they rarely translate to consistent decrements in usable-embryo rate or iDAScore [81]. Oxygen tension, media consistency, and minimization of ROS should be prioritized, and standardized culture handling. Paternal age seldom dictates blastulation once these are optimized [1,21].

#### 5.2.3. Aneuploidy (PGT-A)

When analyses properly control for maternal age and key laboratory covariates, couple-level datasets and donor-oocyte designs consistently show no robust independent effect of paternal age on embryo aneuploidy. Signals that appear in crude or lightly adjusted models are typically small, model-dependent, and not reproducible across cohorts. In practical terms, what looks like a paternal-age effect at first pass usually weakens or disappears once maternal age, stimulation/trigger protocols, insemination method, and culture conditions are in the model.

Reported “paternal-age” trends can hinge on PGT-A platform and calling thresholds, biopsy stage, and clinic-level selection policies. These sources of analytical variance often inflate or obscure small effects and help explain why nominal trends do not persist under rigorous adjustment or external replication. The donor-oocyte literature is particularly informative: with oocyte age constrained, paternal-age contrasts rarely move aneuploidy rates in a clinically meaningful way, reinforcing the conclusion that maternal age dominates this endpoint [2,76].

Sequencing-based donor-oocyte data support this interpretation. In egg-donor cycles with tightly constrained oocyte age, advanced paternal age did not increase embryo aneuploidy rates following blastocyst biopsy. These findings are concordant with other donor-oocyte ICSI series using next-generation sequencing and contrast with earlier small FISH-based reports that suggested higher aneuploidy in men over 50 years [75]. Taken together, these data argue that paternal age primarily influences sperm integrity and epigenetic architecture rather than meiotic chromosome segregation.

Therefore, PGT-A should not be overinterpreted by paternal age. Indications for testing should remain aligned with maternal age, reproductive history, and clinic policy, not used as a surrogate for paternal age. If a paternal-age signal is suspected in a given dataset, interpret it as hypothesis-generating until confirmed under strict covariate control and stable PGT-A pipelines [2,76].

#### 5.2.4. Clinical Pregnancy, Miscarriage, and Live Birth

Large single-center cohorts find no consistent effect of paternal age on clinical pregnancy or live birth once maternal age and laboratory factors are adjusted for [3,81]. Signals can appear only in narrow, stratified contexts. For example, in an IVF-only cohort with normal semen, stratified by maternal age and embryo grade, two findings emerged: (i) higher 2PN rates among fathers < 35 years within the maternal 35–39 years stratum, and (ii) higher miscarriage when paternal age was ≥40 years in maternal < 35 years single-embryo transfers of ≥3AA blastocysts. Importantly, clinical pregnancy and live birth did not differ across paternal-age groups within those strata [87].

In a donor-oocyte cohort (donors ≤ 35 years, with 320 transfers), paternal age showed a graded relationship only with clinical endpoints: the adjusted odds ratio (aOR) for live birth was about 0.50 at 46–50 years and 0.21 at ≥51 years, and miscarriage risk was higher (aOR 2.08) versus ≤35 years. By contrast, fertilization and blastocyst rates did not differ across paternal-age groups [80]. In the same cohort, progressive sperm motility and total sperm count independently predicted embryo quality and clinical outcomes, indicating that age-related motility impairment can act alongside chronological age in shaping donor-oocyte ICSI success [90]. Taken together, these data suggest that any paternal-age impact, when present, is post-fertilization and emerges mainly at very advanced ages, rather than during early morphological milestones [3,80,81,87].

Therefore, for fathers ≥ 46–51 years, a small but plausible increase in miscarriage risk should be included and a reduction in live-birth odds in counseling, while emphasizing that preimplantation endpoints (fertilization, blastulation) are usually stable after adjustment [80].

#### 5.2.5. Molecular Correlates—How Much Reaches the Clinic?

Links between paternal methylation and routine endpoints are modest. In a small IVF series, rDNA-promoter methylation related weakly to 2PN and did not associate with day-3 quality or clinical pregnancy, consistent with locus-specific, low-magnitude signals at fertilization rather than broad embryo-quality shifts [69]. A three-miRNA panel offered modest prediction [43]. Studies incorporating DFI, methylation, and small RNAs sometimes detect associations with embryo competence independent of routine semen parameters—compatible with a chromatin/RNA route rather than gross chromosomal error [5,10,70]. Epigenomic/sncRNA assays should be treated as investigational. None is validated as a stand-alone surrogate of embryo competence. DFI testing should be considered selectively (unexplained ART failure, recurrent early loss, suspected high ROS) and combined with the maternal-age-aware clinical context [1,21,91]. All outcomes from this section are summarized in Table 4.

### 5.3. Counseling and Risk Communication for Couples with Paternal Age ≥ 40–45 Years

Under contemporary IVF and ICSI conditions, the independent contribution of paternal age to fertilization, blastulation, and PGT-A aneuploidy is small after multivariable adjustment and remains modest compared with the maternal component [2,76]. The most plausible routes are epigenomic in nature, including age-linked methylation drift, shifts in nucleosome retention and histone marks, and changes in small RNAs, rather than large effects on aneuploidy [5,10,12,71]. Most donor-oocyte studies show little independent effect of paternal age. One recent series suggests a clinically relevant decline in live birth beyond about 50 years despite similar fertilization and blastulation. Counselors should communicate small to moderate effect sizes in older fathers [80]. In autologous IVF with controlled sperm quality, signals can appear within specific strata. With young maternal age, paternal age of 40 years or more was linked to higher miscarriage after single transfer of high-grade blastocysts, while live birth often remained similar across paternal-age groups. These findings should be treated as context-dependent rather than rigid thresholds [87].

There is no single universally accepted cut-off for “advanced” paternal age, and the relevant threshold depends on whether one focuses on sperm biology or clinical endpoints. Mechanistic and semen studies show that DFI and several epigenetic marks begin to diverge from younger men from the mid-thirties onward, with more consistent shifts after 40 years and steeper changes at very advanced ages, in parallel with oxidative stress and methylation drift [1,5,7,10,12,20,91]. Clinical IVF and ICSI cohorts typically stratify paternal age into bands such as <35, 35–39, 40–44, and ≥45 or ≥50 years when examining fertilization, blastulation, PGT-A aneuploidy, and live birth [2,3,71,76,80,81,82,87]. After multivariable adjustment, fertilization, blastocyst formation, and PGT-A results are largely neutral across these bands, whereas modest increases in miscarriage and reductions in live birth appear only in specific contexts, particularly in donor-oocyte cycles with paternal age ≥ 46–51 years and in young-maternal-age single-embryo transfer strata with paternal age ≥ 40 years [80,82,87].

From a practical standpoint, it is reasonable to frame paternal age of around 40 years as “advanced” for counseling about sperm characteristics and potential ART impact, while emphasizing that the strongest and most reproducible signals relate to very advanced ages (≥50 years) and that maternal age and laboratory factors remain the primary determinants of ART success [2,20,21,76,80].

Time-lapse and AI-based scoring indicate that maternal factors dominate embryo kinetics and grading. Paternal age shows at most a small independent effect under standard laboratory conditions. Couples can be informed that paternal age or BMI may shift early timing slightly, but these magnitudes are modest and not consistently tied to downstream clinical outcomes [78,81]. Sperm miRNA panels currently add only incremental prognostic value, with an area under the curve near 0.70 to 0.75. Results should be interpreted alongside maternal age, ovarian reserve, and laboratory practices, and not used alone for decisions [43].

Reassuringly, obstetric and neonatal endpoints also appear largely neutral with respect to paternal age when maternal age is controlled. In IVF/ICSI pregnancies with autologous oocytes, advanced paternal age was not associated with higher rates of preterm birth, congenital malformations, low birth weight, or adverse perinatal outcomes; only a modest prolongation of gestation was observed in fathers aged 31–40 years compared with those aged 30 [92]. Similar findings have been reported in first frozen-embryo transfer cycles, where paternal age did not significantly affect preterm birth, birth weight, gestational age, or neonatal sex or malformation rates [93]. These data support counseling that focuses paternal-age discussion on implantation and miscarriage risk at very advanced ages rather than on major obstetric or neonatal morbidity.

Therefore, it can be said that risk varies with maternal age, comorbidities, lifestyle, and laboratory quality. Paternal age is best framed as a risk modifier rather than a binary gate [20,21]. Embryo-lineage methylome shifts with advanced paternal age, including neuronal and imprinting enrichment, can coexist with unchanged laboratory endpoints. Any downstream risks are probabilistic and modest, and the discussion should acknowledge uncertainty and selection effects [23].

Observational links exist between advanced paternal age and certain offspring outcomes. Embryo-stage causality through imprinting has not been proven in humans, and age-associated CpG patterns in sperm do not persist after post-fertilization reprogramming. Counseling should be anchored to measurable ART-stage endpoints [17,71].

Actionable points for those 40 to 45 years old and older:

- Modifiable risks before ART: weight, smoking, metabolic health, sleep, and diet patterns that limit oxidative stress [21,22,91].
- DFI should be considered in selected settings such as repeated blastulation failure or early loss, with individualized decisions given mixed evidence [1,71].
- Laboratory practices that minimize reactive oxygen species should be prioritized, consistent culture and ICSI protocols should be maintained, and strategies appropriate for maternal age should be employed. Paternal age rarely changes the first-line approach, but it refines risk framing [2,71,76].

### 5.4. Interventions and Modifiers: Lifestyle, Antioxidants, Timing, Laboratory Practices (Including Sperm Selection)

Higher preconception paternal BMI is associated with differences in early preimplantation development, supporting a modifiable metabolic link on the sperm side [22]. Broader syntheses implicate diet quality, smoking, and environmental pollutants in sperm epigenome perturbations and oxidative stress, which provides a biological rationale to optimize weight, metabolic health, and exposures before ART [21,91]. Because fertilization and blastocyst formation were generally unchanged across paternal age strata, while live birth and miscarriage diverged in older men, the signal aligns more with subtle paternal biology and downstream selection rather than overt preimplantation failure. This supports emphasis on modifiable paternal factors and meticulous laboratory practice rather than age-based changes in treatment algorithms [80].

Given the embryo stage susceptibility of imprinting control regions, workflows should prioritize gentle handling and minimization of oxidative stress, and avoid over-interpreting morphology as a surrogate for imprint fidelity [14]. Antioxidant strategies are mechanistically appealing in the setting of age-related ROS and higher DFI, but clinical effects on hard ART endpoints remain variable. Use should be individualized, time-limited, and paired with management of upstream metabolic contributors, while avoiding polypharmacy [1,21,71].

Outside exceptional circumstances, altering ART timing solely to avoid paternal age has limited empirical support. Priority should be placed on conditioning the modifiable milieu, including weight, smoking cessation, sleep, and exercise, and on ensuring high-quality laboratory practice [21,22].

Minimize oxidative stress from collection through insemination with temperature control, prompt processing, appropriate media, and prudent oxygen exposure, since these factors can amplify age-related damage [21,91]. Evidence on sperm selection is heterogeneous. Benefits, where present, appear context-specific, for example, in high DFI or repeated failure, rather than specific to paternal age. Approaches that enrich for physiologically mature and less damaged sperm can be considered selectively, but current evidence does not support blanket adoption based on paternal age alone. Reporting standards for chromatin-focused assays also require consolidation before routine triage [6,13].

In practice, in couples with advanced paternal age, optimize modifiable risks such as weight, smoking, and metabolic status; reduce oxidative stress at collection and in the laboratory; and consider targeted case-by-case adjuncts such as DFI-informed strategies, rather than paternal age-based changes in the ART algorithm [1,2,6,21,22,76,91]. Actionable counseling points and laboratory levers for APA are outlined in Table 5.

## 6. Beyond ART: Offspring and Long-Term Signals

Advanced paternal age (APA) is linked to small absolute increases in certain neurodevelopmental outcomes, with sporadic perinatal signals that often attenuate after adjustment for parental and socioeconomic covariates [25,26,71]. The most secure pathway is the age-related rise in de novo germline mutations, contributing to rare monogenic disease and a fraction of neurodevelopmental liability [25,26]. Epigenetic routes are mechanistically plausible but appear intergenerational rather than transgenerational: age-DMRs in human sperm do not persist through reprogramming [17], and imprinting reviews note specific paternal contributions without a general age-driven imprinting mechanism [27,28]. Clinically, semen quality/DFI may worsen with age while ART outcomes remain similar across paternal-age strata, implying oocyte-stage buffering [3]. Embryo data show APA-enriched methylation near ASD/SCZ gene sets, but minimal DMR→DEG overlap at blastocyst and null TE pathway signals warrants caution [23].

Interpretation requires methodological vigilance: paternal and maternal ages are correlated. Residual confounding, assortative mating, shared exposures, diagnostic drift, and collider bias can distort estimates [21,24,26,76]. Practical counseling should frame APA as a risk modifier, integrated with maternal age, clinical history, and lab quality, and emphasize modifiable mediators, amenable to intervention before conception/ART [18,19,21]. Frequent yet stochastic embryo-stage imprinting errors (even in high-grade embryos) likely undergo selection, aligning with the low absolute prevalence of imprinting disorders in ART offspring [14]. Cross-species comparisons caution against over-generalization: marmoset vs. human age-linked TSS sets do not overlap and can show opposite trajectories [68].

## 7. Limitations

Evidence on advanced paternal age and preimplantation outcomes relies largely on retrospective or single-center cohorts with limited power, especially at very advanced paternal ages. Residual confounding remains possible because paternal and maternal ages are correlated and because insemination method, culture conditions, oxygen tension, and handling times vary across programs. Assay heterogeneity also constrains inference: DNA methylation platforms, chromatin and DFI assays, and small-RNA pipelines differ in protocols and thresholds, which limits comparability and the derivation of clinically useful cut-offs.

Embryo-level readouts have their own sources of bias. PGT-A findings depend on platform settings, biopsy stage, and mosaicism handling, while time-lapse metrics are sensitive to laboratory workflow. Few studies include matched sperm, embryo, and placenta measures, so mechanistic attribution across stages remains uncertain. Translational generalizability is further limited by species differences in sperm packaging and reprogramming; mechanistic signals defined in mice may not scale to human effect sizes. Finally, although age-linked methylation patterns are reproducible in sperm, persistence into offspring tissues is not observed, which suggests intergenerational rather than stable transgenerational effects.

## 8. Future Perspectives

Several steps could make the evidence more decision-relevant. Prospective, preregistered donor-oocyte cohorts would help isolate paternal contributions, provided they are adequately powered and capture granular laboratory covariates. Harmonized reporting of semen processing and culture conditions, together with standardized protocols for DFI, mitochondrial and oxidative markers, locus-specific methylation, and sperm RNA panels, would improve comparability across centers.

Mechanism-to-clinic studies may benefit from integrating sperm measurements with embryo readouts in matched father–embryo–placenta datasets to examine stage-specific links. Pragmatic interventions that target modifiable paternal factors and laboratory oxidative stress could be tested against both molecular and clinical endpoints, including live birth. Candidate biomarkers, such as sperm epigenetic clocks or focused methylation and small-RNA signatures, would require external validation, calibration, and evaluation of clinical utility before any routine use is considered. Finally, registry-based follow-up that connects ART cycles to perinatal and child outcomes may help quantify absolute risks for counseling and identify subgroups in whom paternal age has greater clinical relevance.

## 9. Conclusions

Maternal age remains the dominant determinant of embryo aneuploidy, yet APA is accompanied by measurable remodeling of the sperm cell. In aggregate, these features support a biologically coherent, intergenerational pathway of methylation, chromatin, embryo signaling, and RNA-mediated effects, most clearly demonstrated in model systems and increasingly suggested in human cohorts.

Clinically, however, the independent impact of paternal age on fertilization, blastulation, morphokinetics, and embryo aneuploidy appears small once maternal and laboratory factors are controlled, and age-linked sperm methylation patterns do not persist across reprogramming into offspring tissues. Therefore, a modest effect size should be communicated. Modifiable mediators (metabolic health, smoking, pollutants, and heat exposures) should be targeted, and oxidative-stress–minimizing laboratory practices should be adopted. With harmonized assays, prospective donor-oocyte studies, mechanistically anchored multi-omics, and carefully designed interventions, the field can move from plausibility to precision, clarifying when, and to what extent, paternal aging meaningfully shifts embryo-stage outcomes and how best to act on that information in ART counseling and practice.

## Figures and Tables

**Table 1:** APA signals in sperm: what changes, how we measure them, and why they matter.

Layer	Main APA Change	Typical Assay	Representative Loci or Markers	Embryo-Stage Link	Evidence Note
DNA methylation	Age-linked CpG drift and DMRs [1,7,29,31]	Arrays, RRBS, capture, targeted qPCR, long-read methods [7,29]	PPARGC1A, RBFOX1, rDNA promoter, imprint-adjacent regions [7,36]	ZGA tuning via methylation→chromatin axis [10]	Human sperm maps and IVF links; mouse causality for methylation, chromatin, embryo [10,39,40]
Nucleosome retention and PTMs	Retention at developmental loci; H3K4 dynamics [39,41]	Calibrated ChIP-seq, CUT&Tag [41]	H3K4me1, H3K4me3 at promoters and enhancers [37,38]	Minor ZGA support; early transcription initiation [37,38]	Mouse mechanistic, human sperm maps [39,41]
Small RNAs	Core sncRNA scaffold with age-responsive peripheral layer [5]	sRNA-seq, RT-qPCR [5]	let-7g, miR-30d, miR-125a-5p, tRF-Gly-GCC, 28S-rsRNAs [11,12,47,48]	Links to embryo quality and cleavage timing; miR-125a-5p→RBM38–p53 [11,12]	Human association and functional models [47,48,50,51]
DNA integrity and ROS	Higher DFI with age and OS signatures [1,54,55]	SCSA, TUNEL, Comet, oxidation assays [55]	DFI, 8-oxoG, lipid peroxidation markers [51,57,58]	Lower fertilization potential and embryo competence [54,55]	Human clinical associations [3,56]
Telomeres	STL rises; LTL shortens [1]	qPCR, TRF [1]	STL, LTL [1]	Offspring LTL tracks with paternal age [53]	Human population links [53]
mtDNA	Higher mtDNA-CN with poorer semen metrics [59,60,61]	qPCR, ddPCR [59]	mtDNA-CN [59]	Motility and concentration shifts [60,61]	Human clinical associations [59]

Overall, aging remodels methylation, chromatin, small RNAs, and integrity. Signals are subtle and probabilistic and act upstream of ZGA and embryo competence.

**Table 2:** Preimplantation outcomes after adjustment for maternal and laboratory factors.

Setting	Paternal Age Bands	Adjusted Endpoints	Direction or Magnitude	Notes on Covariates
Autologous IVF or ICSI	<35, 35–39, 40–44, ≥45 years	Fertilization, blastulation [2,71]	Small or null after adjustment [2]	Strong effects from maternal age and workflow [2,71]
Autologous IVF or ICSI	Same bands	PGT-A aneuploidy [2,15]	No independent effect in most analyses [15]	Maternal age dominates aneuploidy risk [2]
Donor-oocyte cycles	Donors ≤35 years	Blastulation, PGT-A [2,15]	No consistent paternal-age effect [15]	Oocyte age held constant [2,15]
Very advanced paternal age	≥50 years	Fertilization, segmental events [15,75,76,80]	Modest, model-dependent signals [15,80]	Need replication with rigorous modeling [75]
Time-lapse morphokinetics	Continuous paternal age	tPNa to t6, s2, cc2 [77,78,81]	Inconsistent, small shifts [78,81]	Culture, insemination method, media, and maternal factors explain variance [77]
Program-level comparators	By age strata	Usable embryo, blastocyst, clinical, and live birth [3]	No significant differences [3]	Distribution of fertilization methods is similar across age [3]

After controlling maternal and laboratory variables, paternal age has a modest role in fertilization, blastulation, and PGT-A.

**Table 3:** Imprinting and embryo methylome readouts relevant to APA.

Embryo Stage or Tissue	Loci or Feature	Direction and Scope	Pathways or Enrichment	Clinical Implication
Day-3 embryos	SNRPN, KCNQ1OT1, H19 [14]	Frequent hypo and hyper methylation; stochastic [14]	Imprinting control regions [14]	Morphology does not guarantee imprint fidelity [14]
Blastocyst, ICM, and TE	SNRPN, KCNQ1OT1, H19 [14]	Errors remain common; locus-limited [14]	Imprinting and growth pathways [14]	Selection may attenuate risk at birth [14]
Blastocysts with fathers ≥50 y	Genome-scale DMRs in ICM and TE [23]	Global hypermethylation; thousands of DMRs [23]	Neuronal signaling; ASD and SCZ gene sets [23]	KCNQ1OT1 hypermethylation validated in ICM [23]
TE transcriptome	Pathway analysis [23]	No pathway-level disruption in TE [23]	Placental buffering hypothesis [23]	Embryo viability not predicted by TE shifts alone [23]
Intergenerational persistence	Age-DM CpG patterns [17]	Do not persist post-reprogramming [17]	—	Frame as intergenerational and reprogramming-sensitive [17]
Embryo stage or tissue	Loci or feature	Direction and scope	Pathways or enrichment	Clinical implication

In summary, imprinting errors are common and stochastic at the embryo stage. APA links are locus-limited. Stable transgenerational methylation inheritance is not supported in humans.

**Table 4:** Paternal age and ART outcomes from fertilization to live birth.

Endpoint	Unadjusted Signal	Adjusted Signal	Key Modifiers/Confounders	Studies	Clinical Message
Fertilization (2PN)	Mixed, small	Usually non-significant, context-dependent strata exist	Maternal age, IVF vs. ICSI, media/workflow	[2,3,78,81,84,87]	Do not change protocol on paternal age alone; consider ROS/DFIcase-by-case
Blastocyst formation	Small/unstable	Neutral after adjustment	Culture conditions, QA, and insemination method	[2,3,71,81]	Optimize lab handling; paternal age rarely determines yield
PGT-A aneuploidy	Occasional weak trends	No independent effect; maternal age dominates	Maternal age, platform thresholds	[2,76]	Avoid over-interpreting PGT-A vs. paternal age
Morphokinetics	Heterogeneous, subtle	Small adjusted effects, easily confounded	Workflow, media, maternal factors	[2,77,78,81]	Useful for QA/research; not a basis for “age-based” care changes
Clinical pregnancy	Variable	Neutral in most adjusted series	Maternal age, embryo quality protocol	[3,81]	Driven predominantly by maternal and lab factors
Miscarriage	Inconsistent	Increased only in specific strata	Embryo grade, maternal age	[87]	Consider counseling for narrow scenarios; not a broad rule
Live birth	Variable	Neutral overall	Donor design; selection	[3,80]	Possible post-fertilization influence ≥46–51 y; counseling > SOP change

**Table 5:** Counseling and laboratory practice for APA: pragmatic levers.

Lever	What to do	Rationale	When to Consider	Evidence or Notes
BMI and metabolic health	Weight optimization, glycemic control, sleep, exercise [21,22]	Links to sperm epigenome and ROS [21,91]	Men ≥40–45 y and earlier with metabolic risks [22]	Associations with early development timing [22]
Smoking and pollutants	Cessation; reduce PAHs, PM2.5, phthalates, BPA, heat [21,91]	Lowers OS and epigenome perturbation [21]	Pre-ART and during attempts [21]	Mechanistic and clinical syntheses [21]
ROS-minimizing handling	Temperature control; prompt processing; oxygen management; fewer spins [1,64,65,66]	Reduces iatrogenic ROS and DFI [65,66]	All ART cycles [64]	Classic and modern lab data [1,64]
Selective DFI testing	Use in repeated failure or early loss [1,56]	Finite oocyte repair; higher DFI with age [55,81]	Targeted triage, not routine [56]	Mixed outcomes; individualize [55]
Targeted sperm selection	Microfluidics, PICSI, MACS, IMSI when indicated [6,13]	Enrich for mature and less damaged sperm [6]	High DFI or repeated failure [13]	Context-specific benefit only [13]
Antioxidants	Short, individualized courses with lifestyle management [1,21,91]	Addresses ROS biology [21]	Case by case, time-limited [21]	Variable effects on hard endpoints [1]

Overall, APA should be treated as a risk modifier. Prioritize modifiable biology and gentle lab practice. Use targeted adjuncts case by case, not age-based algorithm changes.

## Data Availability

No new data were created or analyzed in this study.
